# Prostate cancer risk assessment and avoidance of prostate biopsies using fully automatic deep learning in prostate MRI: comparison to PI-RADS and integration with clinical data in nomograms

**DOI:** 10.1007/s00330-024-10818-0

**Published:** 2024-07-02

**Authors:** Adrian Schrader, Nils Netzer, Thomas Hielscher, Magdalena Görtz, Kevin Sun Zhang, Viktoria Schütz, Albrecht Stenzinger, Markus Hohenfellner, Heinz-Peter Schlemmer, David Bonekamp

**Affiliations:** 1 Division of Radiology, German Cancer Research Center (DKFZ), Heidelberg, Germany; 2 Heidelberg University Medical School, Heidelberg, Germany; 3 Division of Biostatistics, German Cancer Research Center (DKFZ), Heidelberg, Germany; 4 Department of Urology, University of Heidelberg Medical Center, Heidelberg, Germany; 5 Junior Clinical Cooperation Unit ‘Multiparametric Methods for Early Detection of Prostate Cancer’, German Cancer Research Center (DKFZ), Heidelberg, Germany; 6 Institute of Pathology, University of Heidelberg Medical Center, Heidelberg, Germany; 7 National Center for Tumor Diseases (NCT) Heidelberg, Heidelberg, Germany

**Keywords:** Prostatic neoplasms, Multiparametric magnetic resonance imaging, Risk assessment, Deep learning, Nomograms

## Abstract

**Objectives:**

Risk calculators (RCs) improve patient selection for prostate biopsy with clinical/demographic information, recently with prostate MRI using the prostate imaging reporting and data system (PI-RADS). Fully-automated deep learning (DL) analyzes MRI data independently, and has been shown to be on par with clinical radiologists, but has yet to be incorporated into RCs. The goal of this study is to re-assess the diagnostic quality of RCs, the impact of replacing PI-RADS with DL predictions, and potential performance gains by adding DL besides PI-RADS.

**Material and methods:**

One thousand six hundred twenty-seven consecutive examinations from 2014 to 2021 were included in this retrospective single-center study, including 517 exams withheld for RC testing. Board-certified radiologists assessed PI-RADS during clinical routine, then systematic and MRI/Ultrasound-fusion biopsies provided histopathological ground truth for significant prostate cancer (sPC). nnUNet-based DL ensembles were trained on biparametric MRI predicting the presence of sPC lesions (UNet-probability) and a PI-RADS-analogous five-point scale (UNet-Likert). Previously published RCs were validated as is; with PI-RADS substituted by UNet-Likert (UNet-Likert-substituted RC); and with both UNet-probability and PI-RADS (UNet-probability-extended RC). Together with a newly fitted RC using clinical data, PI-RADS and UNet-probability, existing RCs were compared by receiver-operating characteristics, calibration, and decision-curve analysis.

**Results:**

Diagnostic performance remained stable for UNet-Likert-substituted RCs. DL contained complementary diagnostic information to PI-RADS. The newly-fitted RC spared 49% [252/517] of biopsies while maintaining the negative predictive value (94%), compared to PI-RADS ≥ 4 cut-off which spared 37% [190/517] (*p* < 0.001).

**Conclusions:**

Incorporating DL as an independent diagnostic marker for RCs can improve patient stratification before biopsy, as there is complementary information in DL features and clinical PI-RADS assessment.

**Clinical relevance statement:**

For patients with positive prostate screening results, a comprehensive diagnostic workup, including prostate MRI, DL analysis, and individual classification using nomograms can identify patients with minimal prostate cancer risk, as they benefit less from the more invasive biopsy procedure.

**Key Points:**

- *The current MRI-based nomograms result in many negative prostate biopsies. The addition of DL to nomograms with clinical data and PI-RADS improves patient stratification before biopsy*.
- *Fully automatic DL can be substituted for PI-RADS without sacrificing the quality of nomogram predictions*.
- *Prostate nomograms show cancer detection ability comparable to previous validation studies while being suitable for the addition of DL analysis*.

## Introduction

Recently, multiparametric magnetic resonance imaging (mpMRI) has been established for identification and for targeted prostatic lesion biopsy through cognitive fusion [1], the stereotactic fusion of transrectal ultrasound (TRUS), and MRI [2], or in-bore MRI techniques [3]. With this approach, detection of significant prostate cancer (sPC) and enrollment into active surveillance have improved [4], while the approach promises to spare biopsies altogether in certain men [5, 6]. With growing evidence for the benefits of prostate MRI in prospective multi-center trials [7–9], guidelines increasingly recommend mpMRI prior to biopsy in biopsy-naïve or pre-biopsied men [10]. The decision whom to biopsy can be supported by risk calculators (RCs) incorporating demographic and clinical information such as age, digital rectal exam (DRE), prostate-specific antigen (PSA), and prostate volume, e.g., RCs from the European Randomized Study of Screening for Prostate Cancer (ERSPC) combine this information in logistic regression models [11] and visualize it using nomograms [12]. The positive predictive value (PPV) of clinical radiologist prostate MRI assessment using the prostate imaging reporting and data system (PI-RADS) [13] is limited, variable, and typically reported between 30% and 50% across different centers [14–16], resulting in many negative biopsies. RCs promise to improve patient selection before biopsy, which carries the risk of infection, bleeding, and hospitalization [17]. Recently, it has been shown that RCs benefit from the addition of PI-RADS [18]. Several such RCs have been proposed, none of which exhibits clear advantages over another at the current time [10], while there are concerns about calibration shifts over time and across institutions, reducing their benefits. Simultaneously, fully-automated analysis of prostate MRI using machine learning in the form of deep learning (DL) by convolutional neural networks has recently been demonstrated to provide sPC detection similar to clinical PI-RADS assessment by radiologists [19, 20]. Self-configuring network architectures for semantic image segmentation [21] and object detection [22] can adapt to a wide range of medical imaging modalities. The UNet DL architecture [23] has become especially popular in medical image segmentation [19–21]. Resulting networks indicate the spatial location of identified suspicious findings, allowing comparison to radiologist-identified regions [21, 22]. DL-based prostate MRI assessment carries the potential to make risk assessment tools more reproducible and to foster more widespread application of fully automatic image analysis. We hypothesized that risk estimation with logistic regression models based on demographic and clinical data but with the addition of fully-automated DL image assessment would be capable of performing similarly to previously established risk models using clinical PI-RADS assessment. The goal of our study was to re-evaluate established RCs for prostate cancer risk assessment using a large consecutive cohort from our institution to determine if clinical PI-RADS assessment and fully-automated DL prostate MRI assessment perform similarly in such models, and whether the additional benefit is obtained when both are combined.

## Material and methods

### Study sample

Multiparametric prostate MRI examinations between September 2014 and June 2021 were consecutively included in this retrospective single-center study if patients received combined extended systematic and targeted prostate biopsy and mpMRI at our institutions in Heidelberg, Germany. The institutional ethics committees approved the study and waived informed consent (S-164/2019). Exclusion criteria were (1) prior therapy for PCa (e.g., radiation therapy, ultrasound ablation); (2) previous or ongoing androgen-depriving treatment; (3) severe imaging artifacts; (4) previous prostate biopsy < 2 months ago or interval between MRI and subsequent biopsy > 6 months; and (5) unusually rare histopathology. Exams with any previous diagnosis of prostate cancer, including exams under Active Surveillance, were excluded from the risk calculator evaluation.

### MRI protocol

Multiparametric MRI was performed using two 3.0 Tesla scanners (Megnetom Prisma, Biograph mMR; Siemens Healthineers) and one 1.5 Tesla MRI scanner (Magnetom Aera, Siemens Healthineers) based on PI-RADS recommendations [13, 24] and guidelines of the European Society of Urogenital Radiology [25, 26]. Examinations used the standard multichannel body coil and integrated spine phased-array coil. MRI acquisition parameters are detailed in Supplemental Table 1.

### Biopsy scheme, PI-RADS assessment, and image segmentation

Radiologists assessed mpMRI according to PI-RADS v2.1 guidelines [13] during a clinical routine with access to previous reports, PSA levels, and dynamic contrast-enhanced T1-weighted images (DCE). After the multidisciplinary conference discussion, patients received extended systematic and targeted transperineal MRI/ultrasound-fusion biopsies matching the Ginsburg protocol [27]. For older cases, where only clinical PI-RADS v1 [25] assessments were available, previously-biopsied lesions from the original clinical report were reassessed with PI-RADS v2 by a board-certified radiologist without reviewing the biopsy result and with knowledge of the prior report, thus generating a consensus score using PI-RADS v2 criteria and preserving the relationship between MRI lesion and targeted biopsy result. Biopsies were guided by rigid or elastic software registration. Histopathology was assessed by a dedicated uropathologist with 18 years of experience (A.St.) and graded according to International Society of Urological Pathology (ISUP) standards [28]. sPC was defined as ISUP grade ≥ 2.

### DL model configuration, training, calibration, and inference

Modified nnUNet models were trained for prostate and lesion segmentation with configuration details given in Supplemental Material S-1. Models use whole prostate bpMRI images for predictions and provide nnUNet softmax maps which provide voxel-based tumor probability and thus indicate both, lesion presence and localization. Activation maps were interpreted as the voxel-wise probability of finding sPC, with the maximum probability representing the patient-wise sPC probability score (UNet-probability). The continuous values of the UNet-probability were converted to a 5-point Likert scale (UNet-Likert). UNet-Likert thresholds were dynamically chosen [29] to target sensitivities or specificities similar to PI-RADS, as described in Supplemental Material S-2.

### RCs and decision strategies

Patient age, DRE, PSA, fully automatic T2-weighted segmentation-based prostate volume, and previous biopsy results were available for analysis on each case so that data imputation was not necessary. RCs evaluated included Radtke et al [30], van Leeuwen et al [31], and Alberts et al [32] (MRI-ERSPC). For MRI-ERSPC and Radtke 2017, individual models for biopsy-naïve and previously-biopsied patients were considered. The PI-RADS/PSAD strategy defined low-risk exams as PI-RADS ≤ 2, or PI-RADS 3 with PSA density (PSAD) < 0.1 ng/mL [33] for comparison. To evaluate whether UNet-Likert provides comparable value to clinical PI-RADS assessment, PI-RADS was substituted by UNet-Likert in the RCs (UNet-Likert-substituted RCs), and for comparison in PI-RADS/PSAD (UNet-Likert/PSAD*)*. As PI-RADS and DL may extract complementary information from image data, models combining DL with PI-RADS were calculated (UNet-probability-extended RCs*)*, by estimating two-parameter logistic regression models including the RC score and UNet-probability, similar to the addition of age and PI-RADS to the ERSPC score [32]. Finally, to evaluate the most flexible parametrization, we fitted new multivariate logistic regression models not relying on fixed coefficients or parameter transforms (see Supplemental Material S-3 and Supplemental Table 2). We then used repeated 10-fold cross-validation on the training set to choose the candidate model with the highest cross-validation performance named the newly-fitted PI-RADS *+* UNet probability RC. Table 1 shows the parameters for each model.

**Table 1:** Overview of the existing RCs and the clinical, demographic, and imaging variables used by them

	Age	DRE	PSA	Prostate volume	Biopsy-naïve/prebiopsied	PI-RADS
Radtke 2017	+	+	log (PSA)	+	+	+
Leeuwen 2017	+	+	1/PSA	1/√Volume	+	+
MRI-ERSPC	+	+	log (PSA)	Volume class	+	+
PI-RADS/PSAD			+	+		+
Newly fitted PI-RADS + UNet probability RC	+	+	log (PSA)	1/√(0.01 × volume)	+	+

Note that for PSA and prostate volume, models also differ in their parametric transformations: Radtke 2017 and MRI-ERSPC use a logarithmic rescaling for PSA values. Leeuwen 2017 used the reciprocal of PSA and the reciprocal square root for prostate volume. MRI-ERSPC used three discreet volume classes instead of a continuous scale of volumes for backward compatibility to volume estimations by transrectal ultrasound or DRE. PI-RADS/PSAD describes the strategy of sparing biopsies for exams PI-RADS ≤ 2 or PI-RADS 3 with PSA density < 0.1 ng/mL^2^

Plus signs (+) indicate that the model is relying on the parameter without transformations

*PI-RADS* prostate imaging reporting and data system, *PSA* prostate-specific antigen, *DRE* digital rectal examination

### Statistical analysis

Logistic regression models have been shown to be susceptible to calibration shifts when applied to datasets from new institutions [18, 34], thus, after establishing original and UNet-Likert-substituted model performance, adjustment of these models by intercept-only recalibration (a.k.a. recalibration in the large) and intercept/slope recalibration (logistic recalibration) on the training set was implemented [34–36]. In the UNet-probability-extended RCs, slope, and intercept were necessarily fitted as detailed in Supplemental Material S-4. As Radtke 2017 and MRI-ERSPC consist of separate models for biopsy-naïve or previously-biopsied patients, intercept-only recalibration, intercept/slope recalibration, and UNet-probability extension were done separately for each group. The newly-fitted PI-RADS *+* UNet probability RC is necessarily calibrated to the training data by its derivation. The exam-level predictive performance of Radtke 2017, Leeuwen 2017, and MRI-ERSPC-RCs was evaluated on the test set and compared to the UNet-Likert-substituted RCs, UNet-probability-extended RCs*,* and newly-fitted PI-RADS *+* UNet probability RC. Receiver operating characteristics (ROC) were used to evaluate calibration-independent sPC discrimination with area under the curve (AUC) comparisons. The Brier score was used for combined calibration-dependent evaluation of model calibration and discrimination. Calibration was further assessed using calibration plots and the ratio of average predicted risk to observed sPC rate (Exp/Obs-Ratio). Decision curve analysis (DCA), following the interpretation recommendations by Van Calster et al [37] for the opt-in decision to undergo targeted biopsy, was used to weigh the calibration-dependent benefit of correctly diagnosing a patient with sPC against the harm of over-diagnosing patients without sPC [38], with details given in Supplemental Material S-5. The *p* values were adjusted for multiple testing by Holm–Bonferroni correction. Statistical analysis was performed in R version 4.1.0.

## Results

### Study sample characteristics

**Fig. 1:**
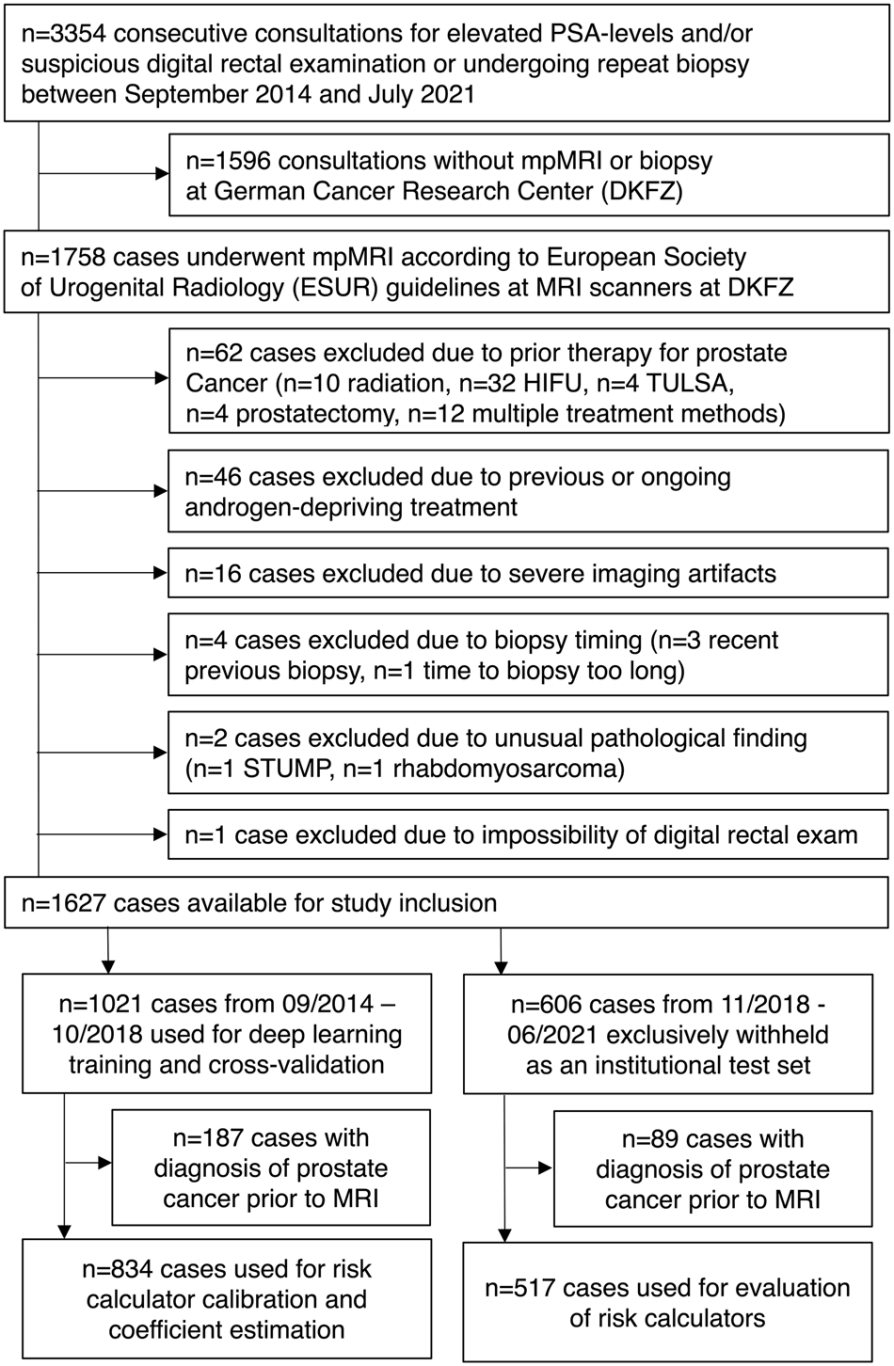
Diagram showing the cohort selection processing with inclusion and exclusion criteria according to STARD guidelines [53]. HIFU, high-intensity focused ultrasound; TULSA, transurethral ultrasound Ablation; STUMP, stromal tumor of uncertain malignant potential

In total, 1627 MRI examinations were included which were temporally split in November 2018 into a training set of 1021 exams, used for DL training in 5-fold cross-validation, and 606 exams for independent testing. 834 exams in the training set and 517 exams in the test set had no previous prostate cancer diagnosis and were used for risk model estimation in cross-validation and subsequent testing. Figure 1 shows inclusion/exclusion criteria as a flowchart. Table 2 gives demographic and clinical characteristics. 1610 exams have been reported in previous publications on DL and radiomics [19, 20, 39], however, data have not been used for systematic clinical RC assessment or development. Regarding the PI-RADS/PSAD strategy, the test set of a previous study overlaps with 101 exams from biopsy-naïve patients with PI-RADS 3 lesions in the current study [33].

**Table 2:** Demographic and clinical characteristics of the training and test cohorts

	Training, (*n* = 834)		Test, (*n* = 517)	
Demographics				
Exams	834	(100)	517	(100)
Age*, y	64	(58–70)	66	(59–72)
PSA*, ng/mL	7.6	(5.3–11.1)	7.2	(5.4–11.0)
Prostate volume*, mL	51	(37–71)	57	(39–78)
PSA density*, ng/mL²	0.14	(0.10–0.22)	0.13	(0.09–0.21)
Suspect DRE	218	(26)	126	(24)
Significant PC (≥ ISUP 2) in systematic/targeted biopsy	321	(38)	200	(39)
No previous biopsy	542	(65)	352	(68)
Previous negative biopsy	292	(35)	165	(32)
PI-RADS, highest lesion per exam				
PI-RADS 1–2	132	(16)	47	(9)
PI-RADS 3	190	(23)	143	(28)
PI-RADS 4	331	(40)	205	(40)
PI-RADS 5	181	(22)	122	(24)
ISUP grade, highest per exam				
No prostate cancer	388	(47)	240	(46)
ISUP grade 1	125	(15)	77	(15)
ISUP grade 2	188	(23)	97	(19)
ISUP grade 3	51	(6)	39	(8)
ISUP grade 4	32	(4)	27	(5)
ISUP grade 5	50	(6)	37	(7)
Location of targeted lesions				
Any location	1540	(100)	918	(100)
Peripheral zone	959	(62)	581	(63)
Transition zone	553	(36)	299	(33)
Multifocal	28	(2)	38	(4)

Values represent a number of cases with percentages of the total shown in parentheses

*PI-RADS* prostate imaging reporting and data system, *ISUP* International Society of Urological Pathology, *IQR* interquartile range, *PSA* prostate-specific antigen, *DRE* digital rectal examination, *PC* prostate cancer

*The median with an inter-quantile range from the 25th to 75th percentile is shown in rows with asterisks

### Imaging-based performance (PI-RADS, DL) and PSA-heuristics

On the test set, clinical PI-RADS achieved 14% [44/317] specificity at 98% [197/200] sensitivity for PI-RADS ≥ 3, and 56% [178/317] specificity at 94% [188/200] sensitivity for PI-RADS ≥ 4. The PI-RADS/PSAD strategy achieved 39% [124/317] specificity at 97% [194/200] sensitivity.

UNet-probability alone achieved an AUC of 0.89 (95% CI: 0.86-0.92). UNet-Likert alone achieved 30% [94/317] specificity at 97% [194/200] sensitivity for UNet-Likert ≥ 3 and 57% [180/317] specificity at 92% [184/200] sensitivity for UNet-Likert ≥ 4. UNet-Likert/PSAD strategy achieved 45% [142/317] specificity at 96% [193/200] sensitivity. UNet-Likert ≥ 3 showed significantly higher specificity compared to PI-RADS ≥ 3 (*p* < 0.001) at similar sensitivities (*p* = 0.32). There was no significant difference in sensitivity or specificity between PI-RADS/PSAD and UNet-Likert/PSAD (*p* = 0.74 and *p* = 0.1, respectively), or between PI-RADS ≥ 4 and UNet-Likert ≥ 4 (*p* = 0.39 and *p* = 0.86, respectively).

### Risk calculator performance (original, UNet-Likert-substituted)

**Fig. 2:**
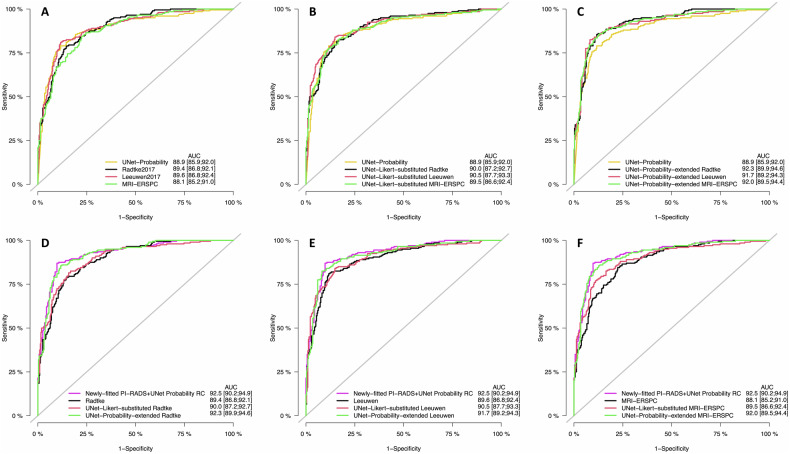
ROCs indicating discrimination ability of RCs. Classification models with no skill would lie on the identity bisector (gray). **A** Original RCs (Leeuwen 2017 (red), Radtke 2017 (black), and MRI-ERSPC (green), compared to UNet-probability (yellow)); **B** UNet-Likert-substituted *RCs* compared to UNet-probability (yellow); **C** UNet-probability-extended RCs compared to UNet-probability (yellow); **D**, **F** differences between original (black), UNet-Likert-substituted (red), and UNet-probability-extended (green) variants of Radtke 2017 (**D**), Leeuwen 2017 (**E**), and MRI-ERSPC (**F**) compared to the newly-fitted PI-RADS *+* UNet probability RC (purple)

Leeuwen 2017, Radtke 2017, and MRI-ERSPC original RCs achieved AUC of 0.90 (95% CI: 0.87–0.92), 0.89 (95% CI: 0.87–0.92), and 0.88 (95% CI: 0.85–0.91), while UNet-Likert-substituted *RCs* achieved 0.90 (95% CI: 0.88–0.93), 0.90 (95% CI: 0.87–0.93), and 0.90 (95% CI: 0.87–0.92), respectively (Fig. 2A, B). There was no significant difference in AUC between the three original RCs (*p* = 0.20) or UNet-Likert-substituted *RCs* (*p* = 0.26) in global testing, so neither Leeuwen 2017, Radtke 2017 nor MRI-ERSPC showed superior AUC. Also, the original and UNet-Likert-substituted versions of each RC showed no significant difference in AUC (*p* = 1.00).

### Risk calculator calibration (original, UNet-Likert-substituted)

**Fig. 3:**
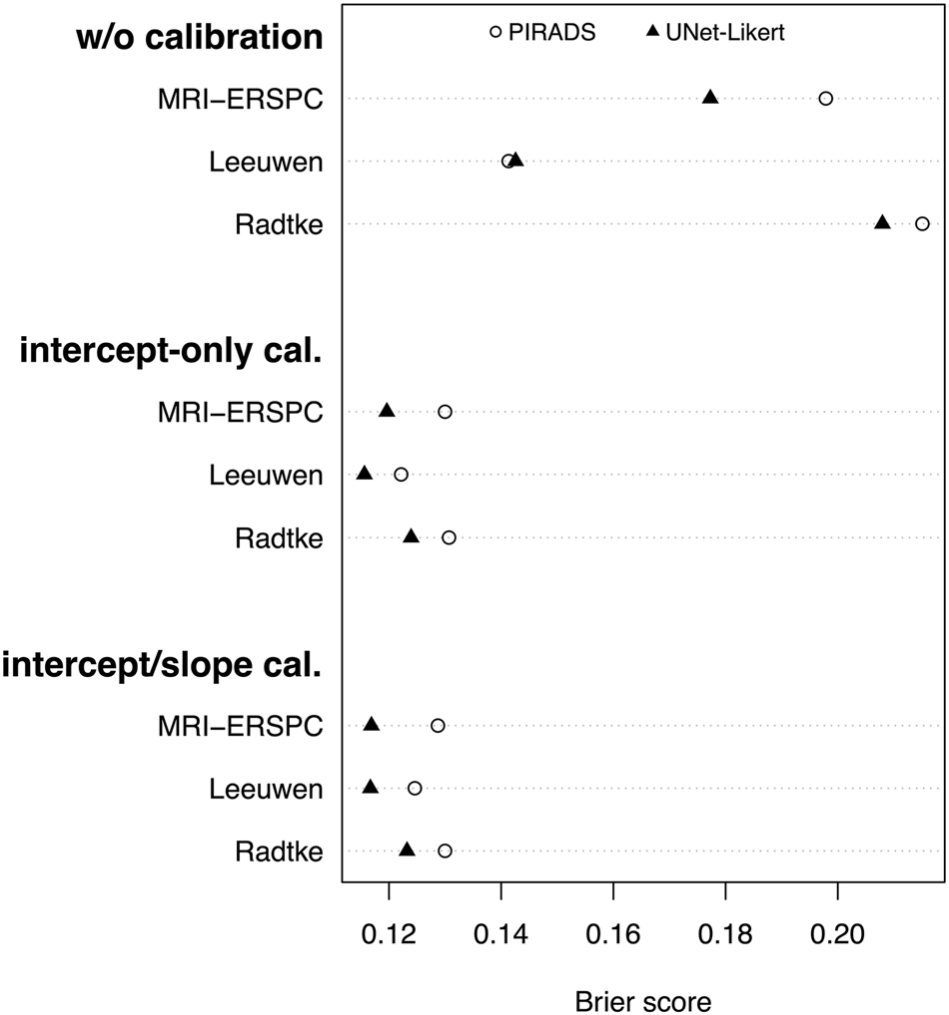
Brier scores, measuring both discrimination and calibration, for original versions w/o recalibration (top), after intercept-only recalibration (middle), and after intercept and slope refitting (bottom). Lower Brier scores indicate better fit. RCs were analyzed with PI-RADS (hollow circle) or UNet-Likert (filled triangle) as their MRI assessment method. As models show no significant difference in discrimination, Brier scores vary mostly due to calibration. Leeuwen 2017 shows the best out-of-the-box calibration, followed by MRI-ERSPC and Radtke 2017. Substitution with UNet-Likert worsens calibration for Leeuwen 2017 and Radtke 2017 slightly but results in an improvement for MRI-ERSPC. After intercept-only calibration, the Brier scores of all models improve drastically, as the worst re-calibrated model has lower scores than the best-uncalibrated model. The effect of UNet-Likert substitution on Brier scores is minimal after intercept-only recalibration. Fitting the model’s slope in addition to the intercept does not lead to improved Brier scores overall and does not decrease Brier score differences between original and substituted models

Original Leeuwen 2017, Radtke 2017, and MRI-ERSPC RCs had Brier scores of 0.14 (95% CI: 0.12–0.16), 0.22 (95% CI: 0.19–0.24), and 0.20 (95% CI: 0.17–0.22), respectively, with Leeuwen 2017 significantly better calibrated than MRI-ERSPC (*p* < 0.001) and Radtke 2017 (*p* = 0.001) (Fig. 3, top, lower is better). After intercept-only calibration, Brier scores of all RCs improved to 0.12 (95% CI: 0.10–0.14, *p* < 0.001), 0.13 (95% CI: 0.11–0.15, *p* < 0.001), and 0.13 (95% CI: 0.11–0.15, *p* < 0.001), respectively (Fig. 3, middle). Intercept/slope recalibration led to only minor statistically insignificant improvements over the intercept-only calibration, at 0.12 (95% CI: 0.11–0.14, *p* = 0.90), 0.13 (95% CI: 0.11–0.15, *p* = 1.00), and 0.13 (95% CI: 0.11–0.15, *p* = 0.61), respectively (Fig. 3, bottom). The Exp./Obs.-Ratio the ratios for the original RCs were 1.29, 1.69, and 0.48, respectively, indicating Radtke 2017 and Leeuwen 2017 overestimated sPC risk while MRI-ERSPC was underestimated. After intercept-only or intercept/slope calibration, Exp./Obs.-Ratio improved to 1.04, 0.99, and 1.02, respectively, with diminishing differences in over- or underestimation between the models. UNet-Likert-substituted *RCs* improved Brier scores for all RCs, however, only the improvement for MRI-ERSPC was statistically significant (*p* < 0.001) (filled triangles in Fig. 3, top). Supplemental Fig. 1 shows calibration plots before and after intercept-only calibration.

### Performance of combined DL and PI-RADS (UNet-probability-extended RCs, newly-fitted PI-RADS + UNet probability RC)

UNet-probability-extended RCs for Leeuwen 2017, Radtke 2017, and MRI-ERSPC achieved AUC of 0.92 (95% CI: 0.89–0.94), 0.92 (95% CI: 0.90–0.95), and 0.92 (95% CI: 0.90–0.94) (Fig. 2C), respectively, and resulted in a significant improvement to the original Radtke 2017 (*p* < 0.001), Leeuwen 2017 (*p* < 0.01), and MRI-ERSPC (*p* < 0.01). The coefficients for UNet-probability-extended RCs are given in Supplemental Table 3.

**Fig. 4:**
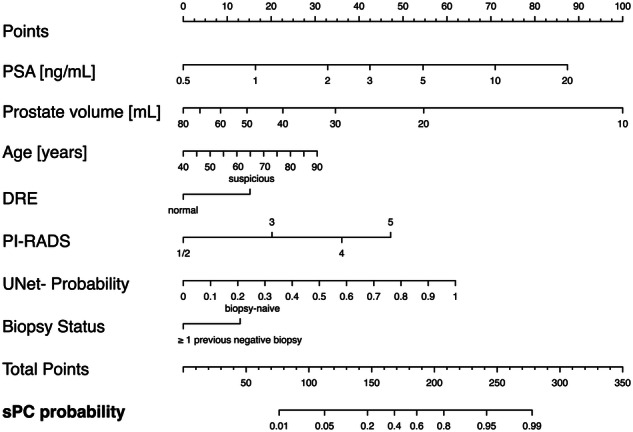
Nomogram for calculating the probability of finding sPC from demographic data, clinical data, radiologist’s mpMRI assessment (PI-RADS), and the predictions of a fully-automated DL system (UNet-probability). Each parameter is projected orthogonally onto the points scale, then points for all parameters are summed. The predicted probability is obtained by orthogonal projection of the total points scale onto the sPC probability scale

The highest test-set cross-validation performance of the candidate models was provided by candidate model #3 which thus was selected as the newly-fitted PI-RADS *+* UNet probability RC (see Supplemental Table 2). Optimal parameters in this model included age, DRE, reciprocal square root of prostate volume, natural logarithm of PSA, biopsy status, PI-RADS score, and UNet-probability resulting in AUC of 0.93 (95% CI: 0.90–0.95) (Fig. 2D–F) and Brier score of 0.10 (95% CI: 0.09–0.12). While the AUC improvements of the newly-fitted PI-RADS *+* UNet probability RC compared to the UNet-probability-extended RCs were not statistically significant (*p* > 0.12), there was a minor improvement. The nomogram for the newly-fitted PI-RADS *+* UNet probability RC is given in Fig. 4, with model parameter significance and odds ratios given in Table 3 indicating the independent contribution of PI-RADS and UNet-probability.

**Table 3:** Odds ratios and model coefficients for each parameter of newly fitted PI-RADS *+* UNet probability RC

Risk factor	Transformation	Coefficient	OR	(95% CI)	*p* value
Age (years)	Linear	0.03	1.03	(1.00–1.06)	0.050
DRE suspicious		0.69	2.00	(1.24–3.22)	0.005
PSA (ng/mL)	${{{{\mathrm{ln}}}}}({PSA})$	1.08	2.95	(2.03–4.28)	< 0.001
Prostate volume (mL)	$\frac{1}{\sqrt{0.01\cdot {volume}}}$	2.23	9.31	(4.60–18.85)	< 0.001
Previous prostate biopsies ≥ 1		−0.59	0.55	(0.35–0.89)	0.015
PI-RADS = 3		0.92	2.51	(0.98–6.41)	0.054
PI-RADS = 4		1.64	5.18	(2.17–12.34)	< 0.001
PI-RADS = 5		2.15	8.61	(3.25–22.81)	< 0.001
UNet-probability from 0 to 1	× 10	0.28	1.33	(1.24–1.42)	< 0.001
(Intercept)		−10.35			

Transformations for prostate volume were suggested by fractional polynomial analysis, where the model versions with the best AUC and Brier score were selected on the training set in repeated 10-fold cross-validation. Odds ratios and their significance levels quantify their influence on sPC predictions. UNet-probability was scaled to 10% increments

*PI-RADS* prostate imaging reporting and data system, *OR* odds ratio, *CI* confidence interval, *PSA* prostate-specific antigen, *DRE* digital rectal examination

### Benefit of avoiding biopsies through risk stratification

**Fig. 5:**
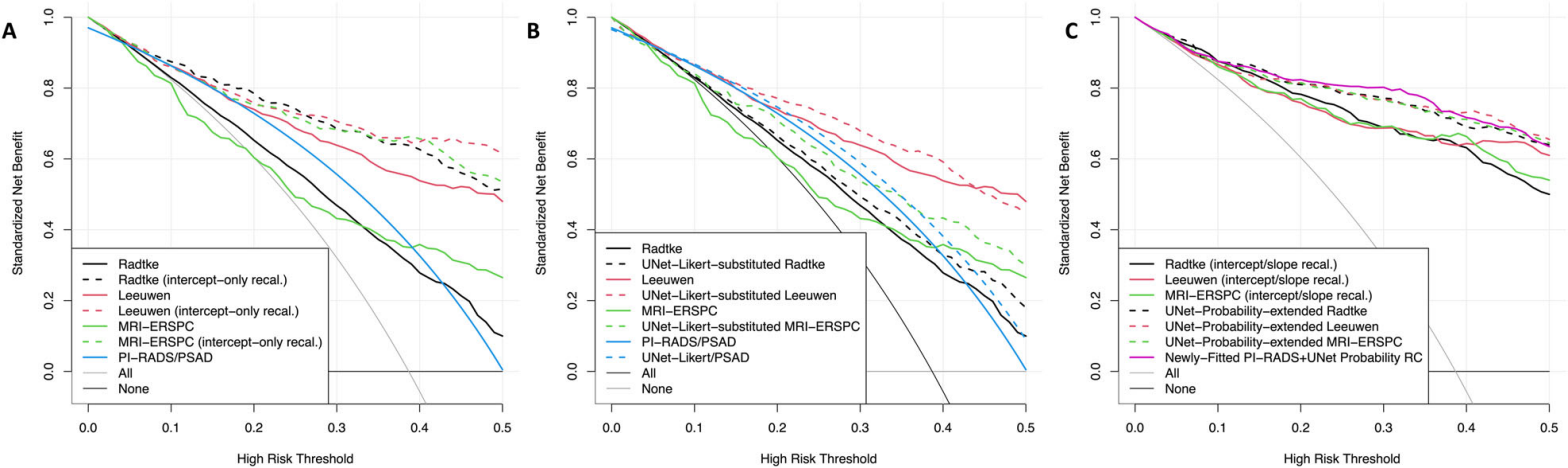
Decision curve analysis comparing existing RCs (Leeuwen 2017 (red), Radtke 2017 (black), and MRI-ERSPC (green)), and the newly-fitted PI-RADS *+* UNet probability RC (purple). The fixed biopsy decision strategy, where the threshold for biopsy is set at PI-RADS 3 with PSAD of ≥ 0.1 ng/mL^2,^ is shown in comparison (blue). **A** Shows the RCs performance as is (solid) or with intercept-only recalibration (dotted), **B** shows each strategy as is (solid) and its UNet-Likert-substituted variant (dotted). Leeuwen 2017 shows achieves a net benefit comparable to the PI-RADS/PSAD strategy and can even exceed it for risk thresholds above 20%, even if manual PI-RADS is substituted with DL image assessment. **C** Shows models after intercept/slope recalibration (solid) and their UNet-probability-extended variants (dotted line) against the newly-fitted PI-RADS *+* UNet probability RC (purple)

Net benefit curves from DCA are given in Fig. 5. Leeuwen 2017 was on par with the PI-RADS/PSAD strategy for thresholds below 20% and showed improved net benefit above that threshold. Radtke 2017 showed benefits against the default strategies in the relevant range while MRI-ERSPC appeared harmful for thresholds below 20% (Fig. 5A). With prevalence adjustment by intercept-only recalibration, Radtke 2017 and MRI-ERSPC compensate for their miscalibration and consequently closely approximate Leeuwen 2017 such that there is no longer a clear benefit for a single RC. UNet-Likert-substituted *RCs* provide higher net benefits than their respective original RCs. UNet-probability-extended RCs improve over the original RCs with intercept/slope recalibration, UNet-Likert-substituted *RC* counterparts, and the PI-RADS/PSAD strategy for risk thresholds above 10%. The newly-fitted PI-RADS *+* UNet probability RC shows further minimal improvement over the UNet-probability-extended RCs, but not over the entire range of relevant thresholds.

**Fig. 6:**
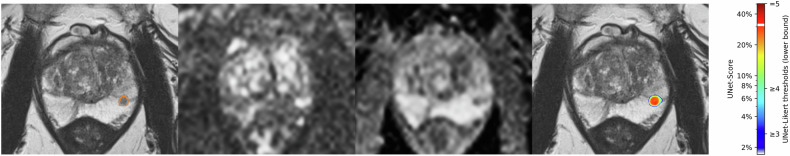
Risk stratification example from the test cohort: a 66-year-old male presents with negative DRE, PSA elevation to 6.2 ng/mL, prostate volume of 82.5 mL, and PSAD of 0.075 ng/mL. The previous prostate biopsy did not find any prostate cancer but MRI uncovers a lesion in the posterolateral peripheral zone with apparent diffusion coefficient restriction, categorized as PI-RADS 4. nnUNet co-detects the lesion and assigns a UNet-probability of 31%, corresponding to UNet-Likert 4. While Leeuwen 2017 and Radtke 2017 RCs follow the PI-RADS recommendation for biopsy with 20% and 45% sPC risk, respectively, the newly-fitted PI-RADS *+* UNet probability RC sets the sPC probability at only 10.5%, below the 15% threshold discussed for biopsy recommendation. MRI-TRUS fusion biopsy did not find any prostate cancer in 30 systematic and nine targeted cores, confirming the newly fitted RCs recommendation to spare the prostate biopsy in this case

Comparison of the absolute number of examinations receiving a recommendation to avoid biopsy by different decision strategies demonstrates that PI-RADS ≥ 4 cut-off spares 37% [190/517] of biopsy sessions at the cost of missing sPC in 12 of 190 spared sessions, which corresponds to a negative predictive value (NPV) of 94%. By adding PSAD through the PI-RADS/PSAD decision strategy, biopsy avoidance is reduced to 25% [130/517] while only 6 sPC are missed in 130 spared biopsies (NPV 95%). The newly-fitted PI-RADS *+* UNet probability RC at the 15% threshold can spare 49% [252/517] of biopsies, 12% more than the PI-RADS ≥ 4 cut-offs (*p* < 0.001) and 24% more than the PI-RADS/PSAD strategy (*p* < 0.001), while missing 16 sPC out of 252 spared biopsies, maintaining the NPV of the PI-RADS ≥ 4 cut-off at 94% (*p* = 0.98) and staying comparable to 95% NPV of PI-RADS/PSAD (*p* = 0.24). These improvements indicate the contributory effect of the UNet-derived information to PI-RADS and clinical information for patient stratification. Table 4 compares the number of biopsies spared by each decision strategy, with the finer histopathological stratification given in Supplemental Table 4. An exemplary case for the clinical benefit of the newly-fitted PI-RADS *+* UNet probability RC at the 15% risk threshold is shown in Fig. 6.

**Table 4:** Comparison of the absolute number of biopsies affected by the decision strategies

	Spared biopsies				Performed biopsies			
	No.	% of all	No. sPC missed	NPV	No.	% of all	No. negative biopsies	PPV
Biopsy none	517	100%	200	61%	0	0%	0	–
Biopsy all	0	0%	0	–	517	100%	317	39%
PI-RADS ≥ 4	190	37%	12	94%	327	63%	139	57%
UNet-Likert ≥ 4	196	38%	16	92%	321	62%	137	57%
PI-RADS/PSAD	130	25%	6	95%	387	75%	193	50%
UNet-Likert/PSAD	149	29%	7	95%	368	71%	175	52%
Newly fitted PI-RADS + UNet probability RC								
10% Risk threshold	193	37%	10	95%	324	63%	134	59%
15% Risk threshold	252	49%	16	94%	265	51%	81	69%
20% Risk threshold	281	54%	21	93%	236	46%	57	76%
25% Risk threshold	301	58%	25	92%	216	42%	41	81%

PI-RADS ≥ 4 recommends biopsies for PI-RADS 4 or PI-RADS 5 exams. PI-RADS/PSAD strategy recommends patients with PI-RADS ≤ 2; and patients with PI-RADS 3 and PSAD < 0.1 ng/mL^2^ to skip biopsy. For the risk model, the threshold value is varied. The absolute number of biopsies avoided by the strategy is shown as “avoided biopsies” with the percentage of the total cohort size given. “No. sPC missed” indicates the number of exams harboring sPC that are recommended to avoid biopsy. “No. negative biopsies” indicates the number of biopsies that are performed without a diagnosis of sPC

*No.* number of exams, *PI-RADS* prostate imaging reporting and data system, *PPV* positive predictive value, *NPV* negative predictive value, *PSA* prostate-specific antigen, *sPC* significant prostate cancer

## Discussion

We find that fully-automated DL biparametric MRI assessment by UNet-Likert scores can substitute for clinical PI-RADS assessment without performance deterioration. After recalibration by adjusting the models’ intercept, all RC models exhibited similar net benefits. Substitution of PI-RADS by UNet-Likert scores demonstrated tendencies for improvement but combining PI-RADS with UNet-probability demonstrated improved discrimination and net benefit, suggesting extraction of complementary information from imaging. Diagnostically important information appears to be present in discordant MRI readings of radiologists and DL. However, DL systems trained with radiologist PI-RADS assessment instead of histopathologically proven sPC as ground truth may not provide similar complementary information. Our results suggest that DL may be able to support diagnostic assessment in settings with limited experience in prostate MRI, as it provided on par performance with experienced radiologists in the current study.

We demonstrated that nearly half of biopsies may spared by the newly-fitted PI-RADS + UNet probability RC while providing an NPV of 94%, which lies above the expected NPV of 86% (95% CI: 0.79–0.91) for mpMRI at this prevalence [16]. Almost doubling the number of spared biopsies comes at the cost of missing 16 sPC cases when using the 15% threshold, compared to six missed sPCs for PI-RADS/PSAD or 12 missed sPCs for PI-RADS ≥ 4. While this increase in false negative cases did not result in a significantly lower NPV, clinicians should critically weigh the decreased morbidity of spared prostate biopsies against the possibility of missing a small number of sPC. For risk-averse patients, establishing a follow-up plan to delay the biopsy instead of avoiding it has the potential to mitigate the consequences of missing sPC and should be investigated further. Being able to quantify and visualize the risk before undergoing an invasive procedure is an additional tool for shared decision-making with the patients about the benefits and harms of the procedure.

The models showed varying degrees of miscalibration when applied to our consecutive test set, but improved substantially by adjusting only the model’s intercept, shown by Brier scores and calibration curves. Leeuwen 2017, the uncalibrated RC from van Leeuwen et al [31], showed the best calibration overall. Leeuwen 2017 and Radtke 2017 slightly overestimated sPC risk while MRI-ERSPC consistently underestimated it before recalibration, which was already observed in previous studies [18, 34, 35, 40, 41] and can reduce their net benefit in DCA. The net benefit of an overestimating model always remains higher than the biopsy-all strategy if the risk threshold remains lower than the cohort prevalence, meaning an overestimating model cannot be harmful at low thresholds. The net benefit of underestimating models approaches the biopsy-none strategy with increasing miscalibration, so they can be harmful to risk thresholds below the cohort prevalence. As current practice favors biopsy for most patients [10], we assume that reasonable risk thresholds lie below the cohort prevalence, so overestimating models (Leeuwen 2017 und Radtke 2017) have an advantage benefit over underestimating MRI-ERSPC ones (MRI-ERSPC), which are potentially harmful in DCA. Deniffel et al [34] raised concerns over the potential miscalibration of RCs and argued that MRI-ERSPC and Radtke 2017 were not clinically useful through DCA. Without calibration, Radtke 2017 showed better clinical utility compared to the default strategies for thresholds over 10% in this study, and Leeuwen 2017 was on par with PI-RADS/PSAD and can even surpass it. With recalibration, all RCs have a higher net benefit than PI-RADS/PSAD.

Our study showed that DL image analysis, PI-RADS, and clinical and demographic parameters have complementary risk prediction values for sPC before biopsy. Predictive performance is also expected to increase with the addition of more risk factors, e.g., free-to-total PSA ratio [42], family history [43], body-mass index [44], or genomic markers [45–48]. DL should be further investigated for clinical decisions after biopsy, as MRI assessments are also a significant predictor for biochemical recurrence and prostatectomy outcomes [48–50].

There are limitations to this study. The RCs shown here are applicable for biopsy-naïve or previously negatively biopsied patients, as these represent the typical screening population. In active surveillance, the use of DL to predict tumor progression risk remains to be investigated [46]. Transperineal MRI/TRUS fusion biopsy provided the reference standard, while prostatectomy specimens would allow for more detailed lesion localization and sPC diagnosis, but prostatectomy cohorts are biased toward sPC-positive cases. As RCs are used in patient stratification before any intervention, the cohort examined in this study much more closely models a typical screening cohort in which an RC would be used, while a prostatectomy cohort would exclude most of the patients who would benefit most from the RC models due to their low-risk status and potential decision to avoid biopsy. The biopsy scheme used in this study has been shown to detect 97% of sPC found at radical prostatectomy [2], providing high-quality ground truth. DL image analysis was performed on bi-parametric MRI, while radiologists interpreted mpMRI including DCE. As the quality of predictions did not decline after PI-RADS was substituted by DL, our data suggest that bi-parametric MRI provides sufficient information. Previous studies showed only minor contributions of DCE to MRI assessment as well [51]. PSA showed a significant contribution to predictions, while PI-RADS and DL image analysis contributed similarly, suggesting that radiologists base their PI-RADS assessment primarily on the mpMRI imaging appearance, although they have access to PSA and PSAD, as intended by PI-RADS [24]. Radiologist-delineated lesions informed the reference standard through targeted biopsy cores while suspicious regions from UNet predictions could not be probed in a retrospective analysis, potentially underestimating the diagnostic potential of DL. However, at our institution, prostate MRI was read by experienced radiologists familiar with subtle manifestations of sPC and frequent review of cases in interdisciplinary boards, and providing high sensitivity at the PI-RADS ≥ 3 threshold of up to 98% compared to extended systematic and targeted biopsies [19]. The ground truth provided by sensitive clinical imaging assessment and a high-quality biopsy scheme leads to an exceptional targeted mapping quality of the prostate while maintaining a clinical workflow. For some of the examinations in the training set, only PI-RADS v1 was available. In these cases, post-hoc reassessment by a board-certified radiologist was performed without knowledge of the biopsy result. By considering the original PI-RADS v1 score, the result was a consensus score, which assured that a representative PI-RADS v2 score was used for training, e.g., a score for which agreement of image characteristics and score were further confirmed. For DL training, this reassessment had only a minor effect as training was based on histopathology from a systematic and targeted prostate biopsy and not PI-RADS, affecting only the ground truth if a lesion was not biopsied by using PI-RADS v1 criteria, which would qualify for biopsy by PI-RADS v2 criteria. As a sensitive approach to prostate assessment was chosen, the probability of this effect with regard to sPC was further minimized. For examinations in the test set, reassessment was not necessary and did therefore not affect performance comparisons. Logistic regression models, as used in the analyzed RCs, enable explainable nomograms but other classifiers should be investigated further, such as support vector machines or random forest classifiers, which have been successfully applied to radiomics [39] and to assess biochemical recurrence [52]. This study retrospectively evaluated cases from a single high-volume tertiary-care center. The benefit of DL-based and updated RCs should be further validated in multi-centric studies.

In conclusion, fully-automated DL prostate MRI assessment not only confirms its similar performance to clinical PI-RADS assessment but also demonstrates complementarity to the latter, with the effect of increased predictive performance of logistic regression risk estimation models utilizing both parameters, thus suggesting an attractive approach for improvement of diagnostic quality for patient stratification before biopsy.

## Supplementary information

Electronic Supplementary Material

## Abbreviations

AUC: Area under the receiver operating characteristics curve
DL: Deep learning
DRE: Digital rectal examination
ISUP: International Society of Urological Pathology
mpMRI: Multiparametric magnetic resonance imaging
PI-RADS: Prostate imaging reporting and data system
PPV/NPV: Positive predictive value/negative predictive value
PSA(D): Prostate-specific antigen (density)
RC: Risk calculator
ROC: Receiver operating characteristics
sPC: Clinically significant prostate cancer
TRUS: Transrectal ultrasound
