# Supplementary material for: Prostate cancer risk assessment and avoidance of prostate biopsies using fully automatic deep learning in prostate MRI: comparison to PI-RADS and integration with clinical data in nomograms
Source: Eur Radiol. 2024 Jul 2;34(12):7909–20. doi: 10.1007/s00330-024-10818-0 (PMC11557625; doi:10.1007/s00330-024-10818-0)
Supplement: Supplementary file 1 — Electronic Supplementary Material [file 330_2024_10818_MOESM1_ESM.pdf]

Prostate Cancer Risk Assessment and Avoidance of Prostate Biopsies using Fully-Automatic Deep Learning in Prostate MRI: Comparison to PI-RADS and Integration with Clinical Data in Nomograms

Electronic Supplementary Material (ESM)

Eur Radiol (2024) Schrader A, Netzer N, Hielscher T et al.

Supplemental Material S-1 Prostate and Lesion Segmentation, and nnUNet Configuration

nnUNet models for prostate segmentation were trained separately for axial T2-weighted images (T2w) and axial low b-value echo-planar imaging DWI (epiDWI) data using manual segmentations that were either created de-novo or edited from proposals of staged nnUNet models that had not been trained with these examples. PCa lesions were retrospectively manually segmented based on descriptions, pictograms, and slice position information given in the clinical reports. Manual segmentations were performed under supervision of a board-certified radiologist fellowship trained in prostate imaging and with 12 years of experience in prostate MRI (*D.B.*).

The self-configuring nnUNet DL architecture [1] was used, as we understood prostate MRI analysis as a semantic segmentation task. Bi-parametric MR data consisting of axial T2-weighted (T2w), high b-value DWI images and apparent diffusion coefficient (ADC) maps were aligned using mutual information co-registration parametrized between T2w and b=0 DWI images. Images were cropped individually to include the whole prostate organ segmentation with additional padding of 15 mm. T2w and high b-value DWI images were normalized separately for each case according to nnUNet standards. Global normalization was applied for ADC, as maps represent physical diffusivity in mm^2/s, which is the same normalization method used by nnUNet for CT images as the latter also already provide a physically meaningful scale. Labels consist of the combined binary lesion segmentation on T2w images, cross-referenced to DWI images. We selected segmentations through the systematically enhanced lesion ground truth method (SELGT) [2]. Segmentations are only included if their targeted biopsy was considered sPC-positive, meaning a sPC-positive core was taken directly from the lesion under MRI guidance or the lesion is in a sextant where sPC was found through systematic biopsy. As nnUNet was primarily developed for anatomy segmentations, we implemented changes to loss functions, learning rate scheduling and oversampling to optimize for prostate lesion segmentation: As prostate lesions make up only a small part of the MRI scan, we implemented focal cross-entropy loss and focal Tversky loss [3] instead of cross-entropy and Dice loss. sPC

cases were oversampled to stabilize training on the class-imbalanced dataset. The learning rate was initially set to 0.001, and then decreased along a cosine anneal function for 1000 epochs with 250 batches per epoch. For inference on the test set, nnUNet test time augmentations, including mirroring, were employed and resulting softmax activation maps for every fold in 2D and 3D were averaged to produce the final activation map. This approach for network training is similar to previous nnUNet adaptations to the task of prostate lesion segmentation with the addition of focal cross-entropy and Twersky loss functions [4]. Deep learning models were trained with PyTorch 1.7.0 and Python 3.9.5 on institutional cluster servers with NVIDIA Titan RTX 24GB cards using driver version 470.57 and CUDA 11.4.

References

1 Isensee F, Jaeger PF, Kohl SAA, Petersen J, Maier-Hein KH (2021) nnU-Net: a self-configuring method for deep learning-based biomedical image segmentation. Nature Methods 18:203-211. doi:10.1038/s41592-020-01008-z

2 Schelb P, Wang X, Radtke JP et al (2021) Simulated clinical deployment of fully automatic deep learning for clinical prostate MRI assessment. Eur Radiol 31:302-313. doi:10.1007/s00330-020-07086-z

3 Abraham N, Khan NM (2019) A Novel Focal Tversky Loss Function With Improved Attention U-Net for Lesion Segmentation2019 IEEE 16th International Symposium on Biomedical Imaging (ISBI 2019), pp 683-687. doi:10.1109/ISBI.2019.8759329

4 Netzer N, Weißer C, Schelb P et al (2021) Fully Automatic Deep Learning in Bi-institutional Prostate Magnetic Resonance Imaging: Effects of Cohort Size and Heterogeneity. Invest Radiol 56:799-808. doi:10.1097/rli.0000000000000791

S-2 UNet-Likert Thresholds and Dynamic Calibration

On the test set, *UNet-Likert* thresholds were dynamically chosen by evaluating the Receiver Operating Characteristics of the preceding 350 exams repeatedly every 50 exams. Note that either sensitivity or specificity can be used to select the thresholds, as the other metric results from the receiver operating characteristics (ROC) of the model. Based on our institutional experience, we empirically calibrated *UNet-Likert* performance using pre-set sensitivities of 100%, 98%, 95%, 90% for *UNet-Likert* 1, 2, 3, 4, respectively, while we calibrated *UNet-Likert* 5 to a specificity of 90%. This method of threshold selection could be scaled up to prospective clinical usage as it only used data available at the time of the MR exam and re-calibration was not needed after every exam. Threshold selection based on a moving window is already established for calibrating machine learning models in prostate cancer [1; 2]. On the training set, *UNet-Likert* scores, for risk model re-calibration and estimation, were determined from the nnUNet model's cross-validation folds with thresholds chosen from ROC analysis on the training set.

References

1 Netzer N, Weißer C, Schelb P et al (2021) Fully Automatic Deep Learning in Bi-institutional Prostate Magnetic Resonance Imaging: Effects of Cohort Size and Heterogeneity. Invest Radiol 56:799-808. doi:10.1097/rli.0000000000000791

2 Schelb P, Wang X, Radtke JP et al (2021) Simulated clinical deployment of fully automatic deep learning for clinical prostate MRI assessment. Eur Radiol 31:302-313. doi:10.1007/s00330-020-07086-z

S-3 Newly-Fitted PI-RADS+UNet Probability Score RC

In repeated 10-fold cross validation, different parameter combinations and transformations were tested for sPC predictions (see Supplemental Table 2). For Candidate model #1, We fitted the default parameter transforms as in Radtke2017. Two additional models using parameter transforms identified by fractional polynomial analysis [1] for separate (Candidate model #2) and pooled (Candidate model #3) modeling of biopsy status were compared. For prostate volume, PSA level and *UNet-Probability*, first-order polynomials and the natural logarithm were investigated as non-linear transformations by fractional polynomial analysis, meaning that for the continuous parameter x, the transformations b x^p with p being chosen from the predefined set [-2,-1,-0.5,0,0.5,1,2,3] were tested for goodness-of-fit [1]. Note that n=-1 corresponds to the reciprocal and n=0.5 to the square root and 0 defines the log-transformation of the input parameter. PI-RADS levels 1 and 2 were combined into a single variable while fitting the logistic regression model, as complete separation was observed for predicting sPC in our data. This is expected as PI-RADS 1 aims to miss no sPC at the expense of very low specificity which makes false negative findings increasingly rare.

References

1 Sauerbrei W, Royston P (1999) Building Multivariable Prognostic and Diagnostic Models: Transformation of the Predictors by Using Fractional Polynomials. Journal of the Royal Statistical Society Series A (Statistics in Society) 162:71-94

S-4 Two-parameter Logistic Regression model for UNet-Probability-extended RCs

The patient-wise probability for finding prostate cancer generated from nnUNet is combined with the original risk model in a new two-parameter logistic regression model with three fitted coefficients: an intercept parameter $\beta_{\text{intercept}}$, an individual slope parameter β_{slope} for the original model and a separate slope parameter $\beta_{\text{UNet-Score}}$ for the newly added *UNet-Probability*, as given by the formula:

$$ln\left(\frac{p}{1-p}\right) = \beta_{\text{intercept}} + \beta_{\text{UNet-Score}} \cdot \text{UNet-Score} + \beta_{\text{slope}} \cdot [\alpha_{\text{intercept}} + \alpha_{\text{age}} \cdot \text{age} + \alpha_{\text{volume}} \cdot \text{Volume} + \ldots]$$

Alpha denotes coefficients from the original RC, Beta denotes newly fitted coefficients and p is the probability of finding sPC. Age and volume are used as examples to illustrate how the original coefficients are included in the new model with the rest of the models parameters and coefficient abbreviated as ellipsis. Note, that extending the model with *UNet-Probability* as an additional parameter in this way includes intercept and slope recalibration. When separate models were available for biopsy-naïve or previously-biopsied patients, they were extended separately.

S-5 Implementation of Decision Curve Analysis

We defined the range of reasonable risk thresholds between 10% (≤ 9 unnecessary biopsies are acceptable per detected sPC) and 50% (≤ 1 unnecessary biopsy is acceptable per detected sPC). Risk thresholds lower than 10% represent a screening context, for which the use of prostate MRI is discouraged in current European guidelines [1]. For risk thresholds under 10%, the benefit of any risk assessment against performing biopsies by default on all patients is expected to become marginal. Settings in which a higher risk threshold may be acceptable could be associated with a patient's desire to avoid invasive biopsies, or individually increased risk for higher rates of complications from anesthesia or infection. In addition, patients may want to avoid the strenuous cycle of repeated negative biopsies resulting from the larger proportion of biopsy indications if lower risk thresholds are used and accept a higher risk of missing sPC. The sPC missed by not operating at very low risk thresholds is expected to be smaller, less aggressive, and less advanced, as finding high Gleason grades at low PI-RADS levels is also rare. The given ranges thus represent clinically realistic scenarios, according to the situation that patients and physicians may not be willing to choose prostate biopsy at any cost, and reflecting the important option of individual risk stratification. An additional factor in the consideration of the risk thresholds used is the available cohort size: the minimum risk threshold that is reasonable to analyze depends on a minimum number of available false negative patients for valid accuracy comparisons within DCA.

References

1 Mottet N, van den Bergh RCN, Briers E et al (2021) EAU-EANM-ESTRO-ESUR-SIOG Guidelines on Prostate Cancer—2020 Update. Part 1: Screening, Diagnosis, and Local Treatment with Curative Intent. European urology 79:243-262. doi:10.1016/j.eururo.2020.09.042

S-6 STARD and TRIPOD checklists

STARD 2015 Checklist

Section & Topic	No.	Item	✔ (N/A)
**TITLE OR ABSTRACT**			
	1	Identification as a study of diagnostic accuracy using at least one measure of accuracy (such as sensitivity, specificity, predictive values, or AUC)	✔
**ABSTRACT**			
	2	Structured summary of study design, methods, results, and conclusions (for specific guidance, see STARD for Abstracts)	✔
**INTRODUCTION**			
	3	Scientific & clinical background, including the intended use and clinical role of the index test	✔
	4	Study objectives and hypotheses	✔
**METHODS**			
Study design	5	Whether data collection was planned before the index test and reference standard were performed (prospective study) or after (retrospective study)	✔
Participants	6	Eligibility criteria	✔
	7	On what basis potentially eligible participants were identified (such as symptoms, results from previous tests, inclusion in registry)	✔
	8	Where and when potentially eligible participants were identified (setting, location and dates)	✔
	9	Whether participants formed a consecutive, random or convenience series	✔
Test methods	10a	Index test, in sufficient detail to allow replication	✔
	10b	Reference standard, in sufficient detail to allow replication	✔
	11	Rationale for choosing the reference standard (if alternatives exist)	
	12a	Definition of and rationale for test positivity cut-offs or result categories of the index test, distinguishing pre-specified from exploratory	✔
	12b	Definition of and rationale for test positivity cut-offs or result categories of the reference standard, distinguishing pre-specified from exploratory	✔
	13a	Whether clinical information and reference standard results were available to the performers/readers of the index test	✔
	13b	Whether clinical information and index test results were available to the assessors of the reference standard	✔
Analysis	14	Methods for estimating or comparing measures of diagnostic accuracy	✔
	15	How indeterminate index test or reference standard results were handled	N/A
	16	How missing data on the index test and reference standard were handled	N/A
	17	Any analyses of variability in diagnostic accuracy, distinguishing pre-specified from exploratory	✔
	18	Intended sample size and how it was determined	N/A
**RESULTS**			
Participants	19	Flow of participants, using a diagram. Include the figure number (preferably figure 1) or page number	✔ Figure 1
	20	Baseline demographic and clinical characteristics of participants	✔ Table 2
	21a	Distribution of severity of disease in those with the target condition	✔
	21b	Distribution of alternative diagnoses in those without the target condition	N/A
	22	Time interval and any clinical interventions between index test and reference standard	
Test results	23	Cross tabulation of the index test results (or their distribution) by the results of the reference standard	✔ Table 4
	24	Estimates of diagnostic accuracy and their precision (such as 95% confidence intervals)	✔
	25	Any adverse events from performing the index test or the reference standard	
**DISCUSSION**			
	26	Study limitations, including sources of potential bias, statistical uncertainty, and generalisability	✔
	27	Implications for practice, including the intended use and clinical role of the index test	✔
**OTHER INFORMATION**			
	28	Registration number and name of registry	N/A
	29	Where the full study protocol can be accessed	N/A
	30	Sources of funding and other support; role of funders	✔

Section/Topic			Checklist Item	Page
**Title and abstract**				
Title	1	D;V	Identify the study as developing and/or validating a multivariable prediction model, the target population, and the outcome to be predicted.	p. 1
Abstract	2	D;V	Provide a summary of objectives, study design, setting, participants, sample size, predictors, outcome, statistical analysis, results, and conclusions.	p. 2
**Introduction**				
Background and objectives	3a	D;V	Explain the medical context (including whether diagnostic or prognostic) and rationale for developing or validating the multivariable prediction model, including references to existing models.	p. 5
	3b	D;V	Specify the objectives, including whether the study describes the development or validation of the model or both.	p. 6
**Methods**				
Source of data	4a	D;V	Describe the study design or source of data (e.g., randomized trial, cohort, or registry data), separately for the development and validation data sets, if applicable.	p. 6
	4b	D;V	Specify the key study dates, including start of accrual; end of accrual; and, if applicable, end of follow-up.	p. 6
Participants	5a	D;V	Specify key elements of the study setting (e.g., primary care, secondary care, general population) including number and location of centres.	p. 6
	5b	D;V	Describe eligibility criteria for participants.	p. 6
	5c	D;V	Give details of treatments received, if relevant.	p. 6
Outcome	6a	D;V	Clearly define the outcome that is predicted by the prediction model, including how and when assessed.	p. 7
	6b	D;V	Report any actions to blind assessment of the outcome to be predicted.	p. 7
Predictors	7a	D;V	Clearly define all predictors used in developing or validating the multivariable prediction model, including how and when they were measured.	p. 7-8 Table 1
	7b	D;V	Report any actions to blind assessment of predictors for the outcome and other predictors.	p. 7
Sample size	8	D;V	Explain how the study size was arrived at.	N/A
Missing data	9	D;V	Describe how missing data were handled (e.g., complete-case analysis, single imputation, multiple imputation) with details of any imputation method.	p.7
Statistical analysis methods	10a	D	Describe how predictors were handled in the analyses.	
	10b	D	Specify type of model, all model-building procedures (including any predictor selection), and method for internal validation.	Supl. Material S-3
	10c	V	For validation, describe how the predictions were calculated.	p. 8
	10d	D;V	Specify all measures used to assess model performance and, if relevant, to compare multiple models.	p. 8-9
	10e	V	Describe any model updating (e.g., recalibration) arising from the validation, if done.	p. 8
Risk groups	11	D;V	Provide details on how risk groups were created, if done.	N/A
Development vs. validation	12	V	For validation, identify any differences from the development data in setting, eligibility criteria, outcome, and predictors.	Table 2
**Results**				
Participants	13a	D;V	Describe the flow of participants through the study, including the number of participants with and without the outcome and, if applicable, a summary of the follow-up time. A diagram may be helpful.	Figure 1
	13b	D;V	Describe the characteristics of the participants (basic demographics, clinical features, available predictors), including the number of participants with missing data for predictors and outcome.	Table 2
	13c	V	For validation, show a comparison with the development data of the distribution of important variables (demographics, predictors and outcome).	Table 2
Model development	14a	D	Specify the number of participants and outcome events in each analysis.	p. 9
	14b	D	If done, report the unadjusted association between each candidate predictor and outcome.	N/A
Model specification	15a	D	Present the full prediction model to allow predictions for individuals (i.e., all regression coefficients, and model intercept or baseline survival at a given time point).	Table 3
	15b	D	Explain how to the use the prediction model.	Figure 4+6
Model performance	16	D;V	Report performance measures (with CIs) for the prediction model.	p. 10-12
Model-updating	17	V	If done, report the results from any model updating (i.e., model specification, model performance).	p. 10-11
**Discussion**				
Limitations	18	D;V	Discuss any limitations of the study (such as nonrepresentative sample, few events per predictor, missing data).	p. 14
Interpretation	19a	V	For validation, discuss the results with reference to performance in the development data, and any other validation data.	p. 13
	19b	D;V	Give an overall interpretation of the results, considering objectives, limitations, results from similar studies, and other relevant evidence.	p.13-14
Implications	20	D;V	Discuss the potential clinical use of the model and implications for future research.	p. 15
**Other information**				

Supplementary information	21	D;V	Provide information about the availability of supplementary resources, such as study protocol, Web calculator, and data sets.	✓
Funding	22	D;V	Give the source of funding and the role of the funders for the present study.	✓

*Items relevant only to the development of a prediction model are denoted by D, items relating solely to a validation of a prediction model are denoted by V, and items relating to both are denoted D;V. We recommend using the TRIPOD Checklist in conjunction with the TRIPOD Explanation and Elaboration document.

Supplemental Table 1. Image acquisition parameters for included MRI exams as indicated by the scanner's DICOM metadata. For T2w echo and repetition times, the 5th to 95th percentile range is shown.

	Modality	Field Strength	Echo Time [ms]	Repetition Time [ms]	In-plane resolution [mm]	Slice thickness [mm]	Field of view [cm]
Training (n=1021)							
Siemens Prisma (n=972)	T2w	3 T	105 - 145	3710 - 9395	0.26 - 0.51	3.0	13.0 - 23.0
	DWI	3 T	48 - 62	3300 - 5900	2.0 - 2.5	3.0 - 3.3	16.4 - 28.0
Siemens Biograph mMR (n=27)	T2w	3 T	143 - 146	7811 - 9521	0.26 - 0.50	3.0 - 3.3	12.8 - 20.0
	DWI	3 T	67 - 91	4900 - 9300	1.0 - 2.2	3.0	19.3 - 28.0
Siemens Aera (n=22)	T2w	1.5 T	123	5610 - 6110	0.57 - 0.60	3.5	22.0 - 23.0
	DWI	1.5 T	68	5300 - 5500	2.6	3.0	29
Test (n=606)							
Siemens Prisma (n=575)	T2w	3 T	96 - 145	3480 - 4230	0.31 - 0.59	3.0	13.0 - 20.0
	DWI	3 T	48-76	3200 - 6100	0.8 - 2.1	3.0 - 4.0	13.0 - 30.0
Siemens Biograph mMR (n=15)	T2w	3 T	91-119	3704 - 8487	0.31 - 0.42	3.0	13.0 - 20.0
	DWI	3 T	68	4800 - 6000	1.0 - 2.0	3.0	20.8 - 28.0
Siemens Aera (n=16)	T2w	1.5 T	100-116	3790 - 5313	0.26 - 0.47	3.0	16.0 - 20.0
	DWI	1.5 T	64 - 68	3800 - 5600	1.2 - 2.3	3.0 - 4.0	24.3 - 28.0

Abbreviations: T2w = T2-weighted; DWI = Diffusion weighted imaging; DICOM = Digital Imaging and Communications in Medicine

Supplemental Table 2. Overview of model and parameter combinations evaluated in repeated 10-fold cross validation for combining *UNet-Probability* and PI-RADS. CV-AUC and CV-Brier represent the averaged AUC and Brier score from cross-validation on the training set. Plus signs (+) indicate that the model is relying on the parameter. For biopsy status, “separate” indicates that two models were fitted, for exams with no previous biopsies (biopsy-naïve) and for exams with at least one negative prostate biopsy in the patient’s history. Pooled models were estimated on both biopsy-naïve and previously biopsied patients, where this biopsy status was used as an additional parameter. Radtke2017, Leeuwen2017 and MRI-ERSPC were extended as described in Supplemental Material S-4. Candidate model #2 and #3 use the parameter transformation for prostate volume suggested by fractional polynomial analysis.

	**Age**	**DRE**	**PSA**	**Prostate Volume**	**mpMRI**	**Biopsy Status**	**CV-AUC**	**CV-Brier**
Candidate model #1	+	+	log(PSA)	+	PI-RADS, UNet-Probability	separate	89.6%	12.0%
Candidate model #2	+	+	log(PSA)	1/√(0.01*Volume)	PI-RADS, UNet-Probability	separate	90.0%	11.8%
Candidate model #3	+	+	log(PSA)	1/√(0.01*Volume)	PI-RADS, UNet-Probability	pooled	90.4%	11.7%
UNet-Probability-extended Radtke	+	+	log(PSA)	+	PI-RADS, UNet-Probability	separate	89.9%	11.8%
UNet-Probability-extended Leeuwen	+	+	1/PSA	1/√Volume	PI-RADS, UNet-Probability	pooled	90.0%	11.9%
UNet-Probability-extended MRI-ERSPC	+	+	log(PSA)	Volume Class	PI-RADS, UNet-Probability	separate	89.4%	12.2%

Abbreviations: CV = cross validation; AUC = Area under the curve; mpMRI = multiparametric magnetic resonance imaging; DRE = Digital Rectal Exam; PSA = Prostate Specific Antigen; ERSPC = European Randomized study of Screening for Prostate Cancer

Supplemental Table 3. Intercept, slope, and UNet-Probability coefficient for UNet-Probability-extended RCs as fitted on the training set. Radtke2017 and MRI-ERSPC were extended separately for biopsy-naïve and previously biopsied exams. Leeuwen2017 used a single model for both groups ("pooled"). Supplemental Materials S-4 describes the process of extending the RCs with UNet-Probability scores in detail.

Risk calculator	Subgroup	$\beta_{\text{intercept}}$	β_{slope}	$\beta_{\text{UNet-Probability}}$
Radtke2017	biopsy-naïve	-2.87	0.83	2.94
	previously biopsied	-2.42	0.97	2.49
MRI-ERSPC	biopsy-naïve	0.00	0.77	3.04
	previously biopsied	-0.50	0.78	2.79
Leeuwen2017	pooled	-1.82	0.5	2.94

Supplemental Table 4. Absolute number cases without prostate cancer, with ISUP 1 or with ISUP ≥2 histopathology that were categorized as low risk by the risk stratification strategies. The Newly-fitted PI-RADS+UNet Probability RC used a threshold of 15% sPC probability.

Risk strategy	No. spared biopsies	no PC	ISUP 1	ISUP ≥2
PI-RADS/PSAD	130	100	24	6
UNet-Likert/PSAD	149	125	17	7
PI-RADS ≥4	190	145	33	12
UNet-Likert ≥4	196	150	30	16
Newly-fitted PI-RADS+UNet Probability RC >15%	252	195	41	16

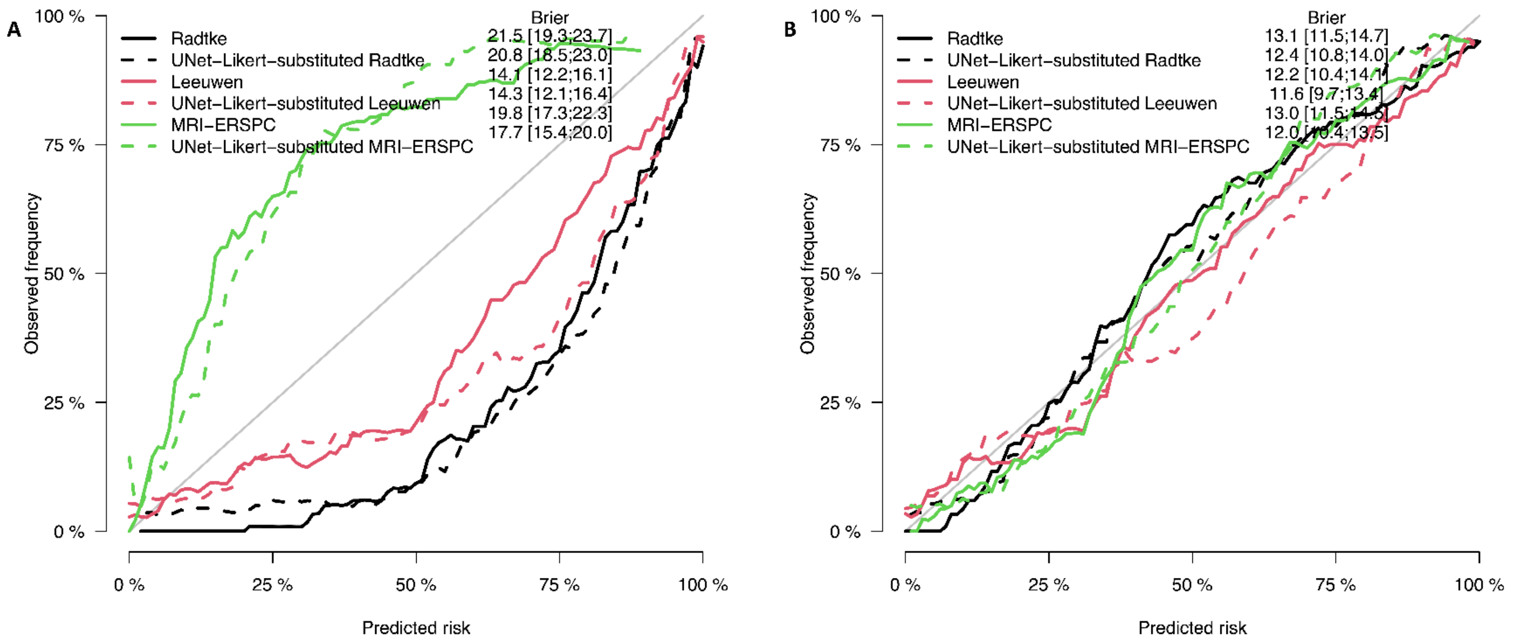

Supplemental Figure 1. Calibration curves showing the estimated risk from the risk calculator on the abscissa and the actual risk for sPC in the test cohort on the ordinate. (A) shows calibration curves from original RCs without re-calibration and (B) shows RCs after intercept-only re-calibration on the training set where model coefficients are preserved. In perfectly calibrated models, the estimated risk and the actual risk will match, and the calibration curve will lie on the bisector (gray). Calibration curves above the bisector indicate that a model is underestimating the actual tumor risk while curves under the bisector indicate overestimating models. Radtke2017 (black) and Leeuwen2017 (red) are overestimating, MRI-ERSPC (green) is underestimating.
